# Safe delivery care practices in western Nepal: Does women’s autonomy influence the utilization of skilled care at birth?

**DOI:** 10.1371/journal.pone.0182485

**Published:** 2017-08-03

**Authors:** Tulsi Ram Bhandari, V. Raman Kutty, P. Sankara Sarma, Ganesh Dangal

**Affiliations:** 1 Department of Public Health, School of Health and Allied Sciences, Pokhara University, Kaski, Nepal; 2 AchuthaMenon Centre for Health Science Studies, Sree Chitra Tirunal Institute for Medical Sciences and Technology, Trivandrum, Kerala, India; 3 Department of Obstetrics and Gynaecology, Kathmandu Model Hospital, Bagbazaar, Kathmandu, Nepal; TNO, NETHERLANDS

## Abstract

Despite various efforts to increase the utilization of skilled birth attendants (SBA), nearly two-thirds of deliveries take place at home without the assistance of SBAs in Nepal. We hypothesized that the ability of women to take decisions about their own lives—women’s autonomy—plays an important part in birth choices. To know this, we conducted a community-based cross-sectional study for assessing women’s autonomy and utilization of safe delivery care service in Kapilvastu district of Nepal from June to October 2014. We used multivariate modeling to associate socioeconomic factors and women’s autonomy with the utilization of safe delivery care services. Just over one-third of women sought institutional delivery care during the birth of their last child. Out of the total deliveries at health facilities, nearly 58% women visited health facility for self-reported emergency obstructive care. Only 6.2% home deliveries were handled by health workers and 14.7% women used the safe delivery kit for home delivery care. Higher levels of women’s education had a strong positive association (odds ratio = 24.11, CI = 9.43–61.64) with institutional delivery care. Stratified analysis showed that when the husband is educated, women’s education seems to work partly through their autonomy in decision making. Educational status of women emerged as one of the key predictors of the utilization of delivery care services in Kapilvastu district. Economic status of household and husband’s education are other dominant predictors of the utilization of safe delivery care services. Improving the economic and educational status may be the way out for improving the proportion of institutional deliveries. Women’s autonomy may be an important mediating factor in this pathway.

## Introduction

Delivery care practices differ with respect to places, countries, and cultures as well as availability and accessibility of the health services[1,2]. Childbirth is a complicated process which is often not under the control of the woman giving birth[3,4]. Delivery care is regarded as safe when attended by skilled birth attendants either at home or in health facilities[3,5].

Globally, one-third of all deliveries were conducted at home without the assistance of skilled health workers in 2008[6].For reducing maternal mortality and morbidity, skilled care at birth is a crucial input[7,8].A systematic review showed that most maternal deaths globally between the year 2000 and 2004 occurred during labor, delivery and early postpartum period. The major causes of death were the obstetric hemorrhage (25%), infections (15%), unsafe abortion (13%), eclampsia (12%) and obstructed labor (8%)[9,10].More than 50% of maternal deaths were directly related to unsafe delivery practice, which is common in developing countries, mostly in Sub-Sahara Africa and South East Asia[11,12].

There is a huge gap in child birth practices between developed and developing countries. In developed countries, most deliveries are attended by skilled birth attendants in the hospital. In South East Asian and Sub-Saharan countries, nearly two-thirds of all deliveries were conducted at home in 2012 without SBAs[13–16].

In Nepal, despite various maternal health care incentives such as conditional cash transfers, free delivery care and cash incentive for health workers, nearly two-thirds of pregnant women (63.1%) delivered their babies at home without skilled health workers between 2006 to 2011.Out of the home deliveries, 6.7% deliveries were assisted by health workers and 5.7% households used safe delivery care kit for home deliveries[17–19].A study from 2000 to 2008 found the use of safe delivery care kit effective in reducing neonatal mortality, sepsis and other postnatal infections in Nepal, India and Bangladesh[18].

Autonomy is an enacted ability of women to influence decision-making, control of physical and financial resources, and freedom of movement[20].Women who had greater autonomy over physical and financial resources were likely to manage their own and children’s health care better and make fertility-related decisions more independently[21,22]. In Nepal, women’s better educational and occupational status and spousal support for seeking care were found to be positively associated with women’s autonomy. Various aspects of women’s autonomy such as the autonomy of movement, economic independence and freedom of communication with the spouse were identified as supportive factors in the utilization of maternal health care services[20,23].

In developing countries, low level of women’s autonomy is associated with the low utilization of maternal health care services as well as poor achievements in maternal health care policies and programs[24–26]. In most of South East Asian countries, women had an inferior position, and less power in decision-making and seeking health care for themselves and their children compared to their male counterparts[23,27]. As a result, women cannot seek required maternal and child health care services without the prior permission of either their husbands or senior members of their family. Women’s autonomy could be a common mediating factor for other socio-economic factors in the utilization of delivery care services. We hypothesized that women who have high autonomy utilize more skilled care at birth than women with restricted or low autonomy.

As one of the signatory members of the Millennium Declaration, Nepal faces the formidable challenge to increase the skilled care at birth and institutional delivery. Most prior studies focused on the roles of socio-economic factors in assessing the utilization of maternal health care services and did not account for the influence of women’s autonomy in the utilization of delivery care services. Hence, we were interested in assessing whether women’s autonomy had a role in influencing the levels of institutional delivery in Nepal

## Materials and methods

We conducted this study in Kapilvastu district of Nepal. It is one of the districts of Western Development Region of Nepal which covers 1738 square kilometers and is situated at the height of 93 to 1491 meters from the sea level. Kapilvastu district had lower utilization of skilled care at birth and other maternal health care services compared with other districts of the western development region and the national average of Nepal in 2012[28] Geographically, this district is situated in Terai and Chure hills where the population comprises predominantly of the ethnic and other disadvantaged groups[19]. We felt that currently available scales to measure women’s autonomy do not suit the local condition fully; hence first we constructed and validated a new scale for measuring women’s autonomy in this region. That was done in Rupandehi district. This work was part of the Ph.D. research of the first author. The Institutional Ethics Committee of Sree Chitra Tirunal Institute for Medical Sciences and Technology, Trivandrum, Kerala, India provided ethical approval for this study “S1 IEC Clearance”. We requested written permission from Public Health Offices of Rupandehi and Kapilvastu districts, the concerned local authorities of Nepal before initiating research. All participants were explained about the objective of the study and their role and rights. We took written informed consent from each respondent collecting their signatures or thumb-prints prior to participating in the study.

### Brief description of scale construction

We conducted a study in Ilaka (a subdivision of district) number eleven of Rupandehi district, Nepal for construction and validation of a women’s autonomy measurement scale. This Ilaka adjoins Kapilvastu district and has similar characteristics where we planned the cross-sectional study. It consists of four village development committees (VDCs). Out of four, we selected two VDCs randomly using lottery method. Based on the ratio of items and respondents (1:10), we administered the scale to 250 married women of reproductive age (15-49years) using convenience sampling method.

During scale construction, first, we defined women’s autonomy (construct) as a capacity of the women to control decision-making, financial and physical resources, and freedom of mobility. We generated the item pool reviewing available literature and prepared a scale which consists of 24 items and covers three dimensions of women’s autonomy, that is, decision making autonomy, freedom of movement autonomy and financial autonomy. Each item is scored zero to two (0- dependent/always, 1- joint/sometime and 2- independent/never), therefore, the possible score has minimum zero to maximum 48. The characteristics of the scale included Cronbach’s Alpha value (0.84), average content validity ratio/ index (0.8) and overall agreement- Kappa value of the items (0.83), which were acceptable [29].

### Study population and data collection

The target population for this study comprised of reproductive age women who had full-term delivery within a year and completed the postnatal period (42 days after childbirth). Sample size was fixed based on the utilization proportion of skilled care at birth (15.92%) of Kapilvastu district in 2012[28], precision = 0.05with assumed design effect = 2and non-response rate = 20% using online OpenEpi statistics software[30]. We selected ten VDCs out of 76 VDCs of the district using a simple random sampling (lottery) method. The number of the sample at VDC level was fixed proportionately based on the population of the VDC. We interviewed 500 women from five electoral constituencies (ECs) and 10 VDCs of the district using a structured interview schedule and the autonomy measurement scale constructed by the authors. With the help of the Female Community Health Volunteers (FCHVs) started in the center of each village and moved in a randomly chosen direction for data collection. We continued household visits clock-wise until obtaining the required number of respondents.

### Study variables and statistical analysis

The subjects of this study were women who delivered at least one child in the last one year preceding the survey. We compared institutional delivery, delivery assisted at home by trained personnel, as well as the use of the safe delivery kit (SDK) against various predictor variables. We used statistical package for the social sciences (SPSS) version 20for analysis[31].

In our multivariate analysis, we chose women’s education, husband’s education and women’s autonomy as key predictors of the use of the facility. We felt that while husband’s education was a major determinant of the economic status of the household, the subject’s score on the autonomy scale reflects her independent decision-making status. Her own educational status represents her awareness in health matters. First, we created two categories for the respondent’s education considering illiterate or less than ten years schooling as ‘less educated’ and ten or more years schooling as ‘educated’. Then, we followed same criteria for husband’s education. For assessing wealth status of the housed we adopted International Wealth Index Scale[32] and the wealth index score was categorized into three i.e. poor, medium class and rich considering tercile. Similarly, we converted women’s autonomy into two categories considering the median value of the total score as the cutoff point. We considered women below the median as ‘with less autonomy’, and median and above the median as ‘with more autonomy’.

We built a binary logistic model with the utilization as the outcome variable and checked for three-way as well as all possible two-ways interactions. A strong interaction was indicated between husband’s education and other variables in the logistic regression model. This indicated that husband’s education modifies the relationship between the woman’s educational status and her autonomy in making the choice to use a facility for delivery. Therefore, we built two separate logistic regression models within the two strata of husband’s education, i.e. illiterate or less than 10 years of schooling and 10 years or more of schooling. Within each, we attempted a mediation analysis i.e. we tried to see whether women’s autonomy acts as a mediating factor on the effect of women’s education in the utilization of safe delivery care services.

## Results

In our sample, 37.7% women sought institutional delivery care for their last childbirth in Kapilvastu district. Almost all institutional deliveries were conducted in governmental health facilities i.e. hospitals (88%), primary health care centers/health posts/sub-health posts (7%) and private hospitals/clinics (5%). Even within the hospital deliveries, nearly six per cent of deliveries were handled by general health workers who were not trained for the skilled delivery care. Out of total institutional deliveries,57.9% women visited health facilities for emergency obstetric care. The emergency obstetric problems were prolonged labor (74.3%), pre-mature labor (8.6%), mal-presentation of the fetus (5.5%). The remaining problems were bleeding before delivery, no fetal movement, retention of urine and anemia.

Of total deliveries, 62.4% were conducted at home. In the home deliveries, only a few women (6.2%) were assisted by health workers. There was low utilization (14.7%) of safe delivery kits for the home delivery. Most of the households used a new-blade and a few households used other unsterilized sharp objects for cutting the umbilical cord of neonates.

Amongst socio-economic factors, women’s education, husband’s education, and husband’s occupation had a strong significant positive association (p<0.001) with the mean score for women’s autonomy. We also found a significant positive association (p<0.01) of the economic status of the household with the mean score of the women’s autonomy. Women’s occupation had no significant association with the mean score of women’s autonomy (Table 1).

**Table 1:** Comparison of socio-economic characteristics of women with their autonomy score.

Predictors (n = 500)	Number of women	Per cent	Mean score of autonomy	F test	P value
Women’s education				34.8	<0.001**
Illiterate	192	38.4	20.6		
< 10 year schooling	150	30.0	22.8		
>10 year schooling	58	11.6	29.7		
Husband’s education				16.2	<0.001**
Illiterate	105	21.0	21.1		
< 10 year schooling	284	56.8	21.3		
>10 year schooling	111	22.2	26.1		
Women’s occupation				1.8	0.15
Agriculture or own business	110	22.0	21.1		
Service	9	1.8	26.2		
Wages or migrant worker	23	4.6	22.3		
Housewife	358	71.6	22.6		
Husband’s occupation				11.7	<0.001**
Agriculture or own business	321	64.2	21.3		
Service	45	9.0	28.7		
Overseas employee	77	15.4	22.4		
Wages or migrant worker	57	11.4	22.9		
Wealth index				5.95	<0.01*
Poor	294	58.8	21.5		
Medium class	184	36.8	23.2		
Rich	22	4.4	26.7		

**F test significant at p < .00 1

*F test significant at p < .01,

The findings show that women with improved socio-economic characteristics were more likely to deliver their child at health facilities compared to their counterparts from low socio-economic status. Women with regular employment were more likely to use institutional delivery care services than housewives, farmers, daily labourers and migrant workers. Women who had better socio-economic status were found more likely to call health worker at home for their delivery care compared to those women who had low socioeconomic status(Table 2).

**Table 2:** Socio-economic characteristics of women and utilization of delivery care services (unadjusted odds ratios).

Variables	Institutional delivery				Home delivery assisted by health workers				Use of SDK for home delivery care			
	Per cent	OR*	95% C.I.^§^ for OR		Per cent	OR	95% C.I. for OR		Per cent	OR	95% C.I. for OR	
			Lower	Upper			Lower	Upper			Lower	Upper
Women’s education												
Illiterate	24.3	Ref.			5.1	Ref.			12.7	Ref.		
< 10 year schooling	42.7	2.3	1.5	3.5	9.3	1.9	0.7	5.0	18.0	1.6	0.8	3.1
>10 year schooling	91.4	33.0	12.7	85	0.0	NA			40.0	4.6	0.7	28.0
Husband’s education												
Illiterate	27.6	Ref.			5.4	Ref.			10.5	Ref.		
< 10 year schooling	32.4	1.3	0.8	2.1	6.4	1.2	0.4	4.0	12.0	1.2	0.5	2.7
>10 year schooling	60.4	4.0	2.2	7.0	6.7	1.3	0.3	5.9	34.1	4.4	1.7	11.0
Women’s occupation												
Agriculture & own business	26.4	Ref.			13.6	Ref.			25.9	Ref.		
Service	66.7	5.6	1.3	23	0.0	NA			66.7	5.7	0.5	66.0
Wages or migrant worker	39.1	1.8	0.7	4.6	14.3	1.1	0.2	5.3	28.8	1.1	0.3	4.0
Housewife	40.2	1.9	1.1	3.1	2.9	0.2	0.1	0.5	8.9	0.3	0.1	0.6
Husband’s occupation												
Agriculture or own business	31.1	Ref.			5.1	Ref.			15.5	Ref.		
Service	84.4	11.7	5.0	27	28.6	7.5	1.3	43.0	0.0	NA		
Overseas employee	35.1	1.2	0.7	2.0	8.2	1.4	0.5	5.4	12.00	0.7	0.3	1.9
Wages or migrant worker	36.1	1.3	07	2.3	5.7	1.1	0.2	5.3	16.7	1.1	0.4	2.8
Wealth index												
Poor	29.3	Ref.			7.4	Ref.			13.9	Ref.		
Medium class	47.7	2.1	1.4	3.1	4.1	0.5	0.2	1.7	16.3	1.2	0.6	1.7
Rich	72.7	6.5	2.4	17.0	0.0	NA			16.7	1.2	0.1	11.0

* odds ratio,

^§^confidence interval

The multivariate models in the two strata show that when the husband is not educated, women’s education is a dominant influence on the choice of institutional delivery. Adding autonomy to the model does not make a difference. On the other hand, when the husband is educated, women’s education seems to work partly through autonomy since by adding autonomy to the model, around 40% [(22.6–13.5)/22.6] of the effects is explained by autonomy (Table 3).

**Table 3:** Women’s education, age and autonomy, and utilization of institutional delivery care services with reference to husband’s education.

Predictors	Model I*			Model II**		
	OR	95% C.I. for OR		OR	95% C.I. for OR	
		Lower	Upper		Lower	Upper
Stratum 1: Husband’s education illiterate or less than ten years schooling						
Women’s education						
less educated	Ref.			Ref.		
Educated	9.7	2.1	45.6	9.1	1.9	42.9
Age of women	1.0	0.9	1.0	1.0	0.9	1.0
Women’s autonomy						
Less autonomous				Ref		
Autonomous				1.3	0.9	2.1
Stratum 2: Husband’s education ten or more than ten years schooling						
Women’s education						
Less educated	Ref.			Ref.		
Educated	22.6	6.3	81.3	13.5	3.5	51.7
Age of women	1.0	0.9	1.1	0.9	0.8	1.0
Women’s autonomy						
Less autonomous				Ref		
Autonomous				6.8	2.3	19.9

* Model I—Women’s education and women’s age

** Model II—women’s education, women’s age and women’s autonomy

## Discussion

The study focused on assessing the role of socio-economic factors, and the extent to which they influence women’s autonomy to utilize safe delivery care services in Kapilvastu district of Nepal. We found a strong positive association with education and women’s utilization of delivery care services. We wanted to assess whether education acts independently or through its effect on women’s autonomy. We found that this pathway differs in the two social strata identified by husband’s education. When husband’s education is low, the odds ratio for women’s education, which was 9.7 in the model without women’s autonomy, is hardly changed (9.1) when autonomy is included in the model. This indicates that in households with low social status, the most important key predictor for improved utilization of institutional delivery care services is the woman’s educational status. Autonomy of the woman hardly seems to make a difference. We can only speculate why this is so. Probably when the economic status is poor, even if there is autonomy, women may have so few life choices that autonomy is not instrumental in bringing about a difference. In the households with high social status indicated by the high level of education for the husband, the odds ratio for women’s education, which is 22.6 in the model without the term for women’s autonomy, reduces to 13.5 when autonomy is introduced into the model. This suggests that around 40% [(22.6–13.5)/22.6], of the effects of women’s education, can be attributed to their autonomy in decision-making. There is more variability in the expression of woman’s autonomy in households of the higher education socioeconomic status.

Thus our major finding is that both the woman’s and the husband’s educational status has the strong positive effect on the use of facility care for delivery, irrespective of whether the woman has decision making power. However, this may give her an additional advantage which cannot be ignored. The concept of autonomy of the woman starts to act only when the socio-economic conditions improve.

Low autonomy of women at the household level is considered as one of the major barriers to increasing the utilization of maternal health care services in Nepal and other developing countries[23,24,33]. In Kapilvastu district, the mean score of the women’s autonomy was 23.34± 8.06 out of the maximum possible score 48. Similarly, we also found that the proportion of the utilization of safe delivery care services was lower in Kapilvastu compared to the national average of Nepal [19].In Nepal, the proportion of skilled care at birth varied the place of residence, socio-economic status, geographical regions, educational status, age and parity of the women[34]. There was significant variation in the utilization of safe delivery care services in terms of couple’s education, husband’s occupation and economic status of households.

Unsafe delivery practice is one of the major causes of maternal mortality in Nepal[35].Hence, the government of Nepal started focused programs such as safe delivery care programmes for promoting safe delivery care throughout the country. The government also formulated National Skilled Birth Attendants Policy, Safe Motherhood, and Neo-natal Health Long Term Plan, National Free Delivery Care Policy from 2000 to 2009 for increasing the utilization of safe delivery care services and assuring the various safe delivery care incentives. Since, 2009, the government integrated different safe delivery care incentives as a safe delivery care package[36–38]. Regardless of various efforts, we found the proportion of home deliveries still high and low utilization of SDK for home deliveries. Both home delivery and use of SDK had the inverse association with women’s autonomy and their socio-economic characteristics.

The study covered only the delivery care practices out of the range of reproductive and maternal health care issues of women in developing countries. The present study was based only on the quantitative dimensions of women’s autonomy as well as the utilization of maternal health care services. Women’s autonomy is a complex subject. It may require more qualitative studies for further precise assessment.

## Conclusions

Low educational status of women is an underlying cause of the low utilization of delivery care services in Kapilvastu district. Economic status of household and husband’s education are other dominant predictors of the utilization of safe delivery care services. Improving the economic and educational status may be the way out for improving the proportion of institutional deliveries, and thereby decreasing maternal and neonatal mortality and morbidity in Kapilvastu district. Women’s autonomy may be an important mediating factor in this pathway.

## Supporting information

S1 IEC Clearance Institutional Ethics Committee (IEC) clearance letter. (PDF)

S1 Data File Survey data for developing women’s autonomy measurement scale. (SAV)

S2 Data File Survey data for assessing women’s autonomy status. (SAV)
