# Supplementary material for: Safe delivery care practices in western Nepal: Does women’s autonomy influence the utilization of skilled care at birth?
Source: PLoS One. 2017 Aug 3;12(8):e0182485. doi: 10.1371/journal.pone.0182485 (PMC5542628; doi:10.1371/journal.pone.0182485)
Supplement: S1 IEC Clearance — (PDF) [file pone.0182485.s001.pdf]

Institutional Ethics Committee (IEC) Clearance Letter

श्री चित्रा तिरुनाल आयुर्विज्ञान और प्रौद्योगिकी संस्थान
तिरुवनन्तपुरम - 695 011, केरल, इंडिया
SREE CHITRA TIRUNAL INSTITUTE FOR MEDICAL SCIENCES AND TECHNOLOGY
THIRUVANANTHAPURAM - 695 011, INDIA
(An Institute of National importance under Govt. of India)

Institutional Ethics Committee
(IEC Regn No. ECR/189/Inst/KL/2013)

SCT/IEC/617/JUNE -2014 11-06-2014

Dr. Tulsi Ram Bhandari
PhD Student
AMCHSS, SCTIMST.

Dear Dr. Tulsi Ram Bhandari,

The Institutional Ethics Committee reviewed and discussed your application to conduct the study entitled "WOMEN'S AUTONOMY AND UTILIZATION OF MATERNAL HEALTH SERVICES IN KAPILVASTU DISTRICT, NEPAL"(IEC/617) on 7th June, 2014.

The following documents were reviewed:

1. *Covering letter.*
2. *Research Proposal.*
3. *Consent form – English & Nepali versions.*
4. *In-depth interview guidelines - English & Nepali versions.*
5. *Structured interview schedule & scale - English & Nepali versions.*
6. *CV – Tulsi Ram Bhandari.*

तार : चित्रमेट
Grams : Chitramet
फोन
Phone : 2443152
फाक्स
Fax : (91)471-2446433
2550728
ई-मेइल
E-mail : sct. @sctimst.ker.nic.in

The following members of the Ethics Committee were present at the meeting held on 7th June, 2014 at G. Parthasarathi Board Room, AMCHSS, SCTIMST.

SL. No.	Member Name	Highest Degree	Gender	Scientific /Non Scientific	Affiliation with Institution(s)
1.	Justice Gopinathan. P.S	BSc. LLB	Male	Legal Expert (Chairperson)	*No*
2.	Dr. Meenu Hariharan	DM	Female	Clinician (Gastro Enterologist)	*No*
3.	Dr. M.D. Gupte	MD, DPH	Male	Public Health	No
4.	Dr. R.V.G. Menon	PhD	Male	Lay Person	No
5.	Dr. Mala Ramanathan	MSc, PhD, MA	Female	Ethicist/Social Scientist (Member Secretary)	Yes

IEC Decision

The IEC approved the conduct of the study in the present form.

Remarks:

The Institutional Ethics Committee expects to be informed about the progress of the study, any SAE occurring in the course of the study, any changes in the protocol and patient information/informed consent and asks to be provided a copy of the final report.

There was no member of the study team /guide who participated in voting / decision making process. The ethics committee is organized and operated according to the requirements of Good Clinical Practice and the requirements of the Indian Council of Medical Research (ICMR).

Sincerely,

MRamanathan

Mala Ramanathan
Member Secretary, IEC
